# Diversity in Mission Statements and Among Students at US Medical Schools Accredited Since 2000

**DOI:** 10.1001/jamanetworkopen.2023.46916

**Published:** 2023-12-14

**Authors:** Kelsey West, Leen Oyoun Alsoud, Kathryn Andolsek, Sara Sorrell, Cynthia Al Hageh, Halah Ibrahim

**Affiliations:** 1 Indiana University School of Medicine, Indianapolis; 2 Department of Medicine, Khalifa University College of Medicine and Health Sciences, Abu Dhabi, United Arab Emirates; 3 Department of Family Medicine and Community Health, Duke University School of Medicine, Durham, North Carolina; 4 Department of Emergency Medicine, Indiana University School of Medicine, Indianapolis

## Abstract

**Importance:**

Diversity in the physician workforce improves patient care and decreases health disparities. Recent calls for social justice have highlighted the importance of medical school commitment to diversity and social justice, and newly established medical schools are uniquely positioned to actively fulfill the social mission of medicine.

**Objective:**

To identify diversity language in the mission statements of all medical schools accredited since 2000 and to determine whether the presence of diversity language was associated with increased diversity in the student body.

**Design, Setting, and Participants:**

Cross-sectional study of public websites conducted between January 6, 2023, and March 31, 2023. Qualitative content analysis of mission statements was conducted using a deductive approach. Eligible schools were identified from the 2021-2022 Medical School Admission Requirements and American Medical Colleges and American Association of Colleges of Osteopathic Medicine websites. Each school’s publicly available website was also reviewed for its mission and student body demographics. All United States allopathic and osteopathic medical schools that have been accredited and have enrolled students since 2000.

**Exposure:**

Content analysis of medical school mission statements.

**Main Outcomes and Measures:**

Prevalence of diversity language in medical school mission statements and its association with student body racial diversity. Data were analyzed in 5-year groupings: 2001 to 2005, 2006 to 2010, 2011 to 2015, and 2016 to 2020).

**Results:**

Among the 60 new medical schools (33 [55%] allopathic and 27 [45%] osteopathic; 6927 total students), 33 (55%) incorporated diversity language into their mission statements. In 2022, American Indian or Alaska Native individuals accounted for 0.26% of students (n = 18), Black or African American students constituted 5% (n = 368), and Hispanic or Latinx individuals made up 12% (n = 840). The percentage of schools with diversity language in their mission statements did not change significantly in schools accredited across time frames (60% in 2001: mean [SE], 0.60 [0.24] vs 50% in 2020: mean [SE], 0.50 [0.11]). The percentage of White students decreased significantly over the time period (26% vs 15% students in 2001-2005 and 2016-2020, respectively; *P* < .001). No significant differences were observed in student body racial or ethnic composition between schools with mission statements that included diversity language and those without.

**Conclusions and Relevance:**

In this cross-sectional study of US medical schools accredited since 2000, diversity language was present in approximately half of the schools’ mission statements and was not associated with student body diversity. Future studies are needed to identify the barriers to increasing diversity in all medical schools.

## Key Points

**Question:**

What is the prevalence of diversity language in mission statements of US medical schools established after 2000, and is diversity language associated with increased student body diversity?

**Findings:**

In this cross-sectional study of 60 new medical schools, 33 (55%) referenced diversity language in their mission statements; there was no significant change in the presence of such language in schools established over the past 20 years. Student body diversity was not significantly different between schools with diversity language and those without.

**Meaning:**

These results suggest that more efforts are needed to improve diversity in the national medical student body to fulfill the social mission of medical education.

## Teaser

This cross-sectional study evaluates whether the presence of diversity language in the mission statements of medical schools accredited since 2000 is associated with increased racial and ethnic diversity in the student body.

## Introduction

The past 2 decades have witnessed increasing calls for equity and social justice in the US health care system. The COVID-19 pandemic exposed stark disparities, with racial and ethnic minority individuals experiencing higher rates of severe illness and death.^1^ While several studies have documented an already higher risk of maternal complication and mortality rates for pregnant Black women,^2,3^ the *Dobbs, State Health Officer of the Mississippi Department of Health, et al v Jackson Women’s Health Organization et al*^4^ Supreme Court ruling has created additional limitations on access to reproductive health care in underserved communities.^5^ Moreover, the court’s recent decision regarding affirmative action will likely adversely affect the care provided to marginalized communities by compounding the existing barriers faced by individuals historically underrepresented in medicine (URM; individuals from racial and ethnic backgrounds who are underrepresented in medicine relative to their prevalence in the general population, namely Black or African American, Hispanic or Latinx, American Indian or Alaska Native, and Native Hawaiian or Other Pacific Islander), as similar state laws have already negatively impacted their entry into public medical schools.^6^

Diversifying the US physician workforce has become a national imperative. Improving congruence between the US population and its physician workforce is necessary for advancing health care quality and mitigating health inequities.^7^ Physician diversity is associated with improved patient satisfaction, engagement, and treatment adherence.^8,9^ Increasing diversity among physicians broadens access to care, decreases health disparities, and improves health outcomes^10^; URM physicians are more likely to pursue primary care specialties and serve in medically underserved communities.^7,11,12^ The inclusion of URM students in medical education also positively influences the future care provided by non-URM physicians. As medical schools serve as the pathway to future physicians, the first step to achieving a diverse physician workforce is ensuring diversity within the national medical student body. However, the recruitment of URM students in medicine has stalled over the past few decades, with worrisome setbacks in states that have implemented race-neutral admission policies.^6,13^ The slow progress has been attributed to historical power structures and deeply rooted beliefs and practices in medical schools that have mitigated the impact of diversity initiatives,^14^ with several studies^14,15^ highlighting entrenched cultural resistance to change in medical schools.

Mission statements have often been viewed as a school’s public commitment to societal needs. They highlight the purpose and priorities of an institution and can influence resource allocation.^16,17,18^ Prior studies of medical school mission statements have revealed traditional themes, such as education, research, and service, while diversity and social justice are less commonly emphasized.^18,19^ Mission statements also evolve over time, with recently accredited schools more likely to include language related to diversity and equity.^19^ Yet, despite their ubiquity, mission statements have rarely been directly linked to outcomes.^20^ The purpose of this study was to investigate the association between the prevalence of diversity language, including terms like *diversity and inclusion*, in the mission statements of US medical schools established between 2000 and 2022 and the racial and ethnic composition of their student bodies. We hypothesized that the newly established schools would be less burdened by institutional structures that maintain the status quo and, thereby, have a greater emphasis on diversity in their mission statements and a more diverse student population. Although we recognize that race and ethnicity are just a few measures of the diversity of medical students, we used race and ethnicity data because they are more consistently reported by medical schools.

## Methods

Between January 6, 2023, and March 31, 2023, we conducted a cross-sectional study of mission statements and student body characteristics of newly accredited US medical schools using information publicly available on the internet or published databases. We received an ethics exemption from the Khalifa University Institutional Review Board as the study did not involve human participants. This study followed the Strengthening the Reporting of Observational Studies in Epidemiology (STROBE) reporting guideline.

### Setting and Eligibility

Medical school growth has no predictable pattern. The number of medical schools increased dramatically in the 1960s and 1970s. Yet, no new allopathic schools and only 4 osteopathic schools were established in the following 3 decades. Fearing a looming physician shortage, the Association of American Medical Colleges (AAMC) called for a nationwide expansion in medical schools in 2006, and 60 new schools were established and accredited between 2001 and 2022.^21,22^

We considered all US medical schools established from 2000 on, during the most recent wave of medical school expansion, to be new medical schools. Allopathic and osteopathic schools that achieved initial Liaison Committee on Medical Education or American Osteopathic Association Commission on Osteopathic College Accreditation accreditation and enrolled students between January 1, 2000, and December 31, 2022, were included. Schools currently in development or existing schools that had not been accredited or enrolled a first class of students were excluded.

### Data Collection

Eligible schools were identified and data were collected from the 2021-2022 Medical School Admission Requirements,^23^ provided by the AAMC,^24^ and the American Association of Colleges of Osteopathic Medicine.^25^ Race and ethnicity classifications included American Indian or Alaska Native; Asian; Black or African American; Hispanic or Latinx; Native Hawaiian or Other Pacific Islander; White; and other, which included multiple race or ethnicity and individuals who did not disclose race information. Schools established after 2020 were excluded due to incomplete student body demographic data available on websites. Racial categories are in accordance with guidelines provided by the US Office of Management and Budget and the US Census Bureau, and these data are based on self-identification. Mission statements were obtained from each school’s website. Two researchers (K.W. and L.O.A.) independently extracted student demographics information from each school. Discrepancies were resolved by consensus.

### Coding and Reflexivity

We conducted a literature review^16,17,18,19,26^ to identify language promoting social justice, diversity, and inclusivity in mission statements of medical schools and public health universities and developed a codebook using these terms (eg, *cultural competence* and *social responsibility*). Coding was an iterative process, with regular discussions among the research team, and new codes were added as needed (eTable in Supplement 1). Content analysis of each medical school’s current mission statement was independently conducted by 2 researchers (H.I. and S.S.) in June and July 2023. Using a deductive approach, line-by-line coding was manually performed to identify terms in the constructed codebook. Discrepancies were resolved through consensus and, when needed, with a third reviewer (K.A.).

The authors have diverse racial, cultural, and educational backgrounds. Team members include a current medical student (K.W.) and clinician-educators involved in undergraduate and postgraduate medical education (K.A., H.I., and S.S.). The all-female team has several members who completed medical training in the US decades prior to medical school enrollment reaching gender parity. We reflected upon how our personal experiences of academic medicine influenced our analysis and interpretation of the data.

### Statistical Analysis

Descriptive analysis was performed using R software version 4.2.2 (R Project for Statistical Computing). χ^2^ Tests were conducted to assess the association between race and ethnicity and different school characteristics (public vs private, osteopathic vs allopathic) and across the time periods (2001-2005, 2006-2010, 2011-2015, and 2016-2020). This time frame was selected because it represented at least 1 graduating class, as the AAMC reports 5-year graduation rates for medical schools.^27^ Our objective was to examine schools established within each period to identify emerging patterns in the later-established schools (from 2016 on). A bar plot was generated using Excel version 16.7 (Microsoft Corp) to visualize the distribution of race and ethnicity across different time periods, representing the frequency of each racial and ethnic group within the categorical variable. A 2-sided *P* < .05 was considered statistically significant.

## Results

Of the 60 new medical schools (33 [55%] allopathic and 27 [45%] osteopathic; 6927 total students) that received accreditation and enrolled students since 2000, 33 (55%) included diversity language in their mission statement. Of the 60 schools, 14 (23%) mission statements referred to diversity in the context of their students or future workforce, and 27 (45%) referred to diverse patient populations. The percentage of schools that included diversity language in their mission statements did not statistically significantly change over the 20-year time period (60% in 2001: mean [SE], 0.60 [0.24] vs 50% in 2020: mean [SE], 0.50 [0.11]).

The Table displays the racial and ethnic characteristics between schools that contained diversity language in their mission statement compared with the schools that did not. Overall, there were no significant differences in the racial and ethnic composition of student bodies in schools whose mission statements referenced diversity compared with those that did not (American Indian or Alaska Native: 0 vs 0 in those without and with diversity language; Asian: 44% vs 56%; Black or African American: 38% vs 62%; Hispanic or Latinx: 53% vs 47%; Native Hawaiian or Other Pacific Islander: 0 vs 0; White: 46% vs 54%; and other: 47% vs 53%). No significant differences were found between schools that referenced diversity and those that did not in allopathic, public, or private schools.

**Table.** Racial and Ethnic Characteristics Between Schools That Contained Diversity Language in Their Mission Statement Compared With the Schools That Did Not

Race and ethnicity^a^	Students per school without diversity language in mission statement, mean, No. (%)	Students per school with diversity language in mission statement, mean, No. (%)	*P* value
All schools			
American Indian or Alaska Native	0	0	
Asian	28 (44)	36 (56)	.31
Black or African American	5 (38)	8 (62)	.40
Hispanic or Latinx	16 (53)	14 (47)	.71
Native Hawaiian or Other Pacific Islander	1 (100)	0	NA
White	58 (46)	68 (54)	.37
Other^b^	8 (47)	9 (53)	.80
Allopathic schools			
American Indian or Alaska Native	1 (100)	0	.31
Asian	24 (48)	26 (52)	.77
Black or African American	5 (36)	9 (64)	.28
Hispanic or Latinx	16 (55)	13 (45)	.57
Native Hawaiian or Other Pacific Islander	0	0	NA
White	35 (45)	42 (55)	.42
Other^b^	7 (54)	6 (46)	.38
Osteopathic schools			
American Indian or Alaska Native	0	0	NA
Asian	37 (47)	42 (53)	.57
Black or African American	6 (46)	7 (54)	.78
Hispanic or Latinx	16 (52)	15 (48)	.85
Native Hawaiian or Other Pacific Islander	0	0	NA
White	110 (56)	86 (44)	.06
Other^b^	11 (61)	7 (49)	.34
Public schools			
American Indian or Alaska Native	1 (100)	0	.31
Asian	22 (44)	28 (56)	.39
Black or African American	5 (36)	9 (64)	.28
Hispanic or Latinx	9 (35)	17 (65)	.11
Native Hawaiian or Other Pacific Islander	0	1 (100)	.31
White	35 (47)	40 (53)	.56
Other^b^	5 (25)	15 (75)	.99
Private schools			
American Indian or Alaska Native	0	0	NA
Asian	31 (44)	40 (56)	.29
Black or African American	5 (42)	7 (54)	.56
Hispanic or Latinx	21 (62)	13 (43)	.17
Native Hawaiian or Other Pacific Islander	0	0	NA
White	72 (47)	82 (51)	.42
Other^b^	10 (50)	10 (50)	.99

Abbreviation: NA, not applicable.

^a^ Racial categories are in accordance with guidelines provided by the US Office of Management and Budget and the US Census Bureau; these data were based on self-identification.

^b^ Other included students who identified as multiple races or ethnicities and students who did not disclose race information.

In the 55 of the newly established medical schools that provided data on race and ethnicity, in 2022, American Indian and Alaska Native individuals accounted for 0.26% of students (n = 18), Black or African American students constituted 5% (n = 368), and Hispanic or Latinx individuals made up 12% (n = 840). These numbers fall well below the groups’ representation in the overall US population, which was 0.7% for American Indian or Alaska Native individuals, 13% for Black or African Amerian individuals, and 19% for Hispanic or Latinx individual in 2022.^28^ In comparison, in the 2022-2023 AAMC report of enrolled medical students, American Indian or Alaska Native students constituted 0.3%, Black or African American students made up 8%, and Hispanic or Latinx individuals accounted for 12% of students in 2022.^29^

The Figure illustrates the percentage of schools were established in each of the 5-year intervals that incorporated diversity language in their mission statements alongside the racial and ethnic composition of their student bodies within the same time frames. The percentage of institutions including diversity terms in their mission statements was not significantly different from that in schools that did not when comparing universities established in the 2001-2005 time period (60%) and those established in the periods of 2006-2010 (67%), 2011-2015 (44%), and 2016-2020 (50%). However, we found that the percentage of White students decreased significantly over the time period (26% vs 15% students in 2001-2005 and 2016-2020, respectively; *P* < .001).

**Figure.**
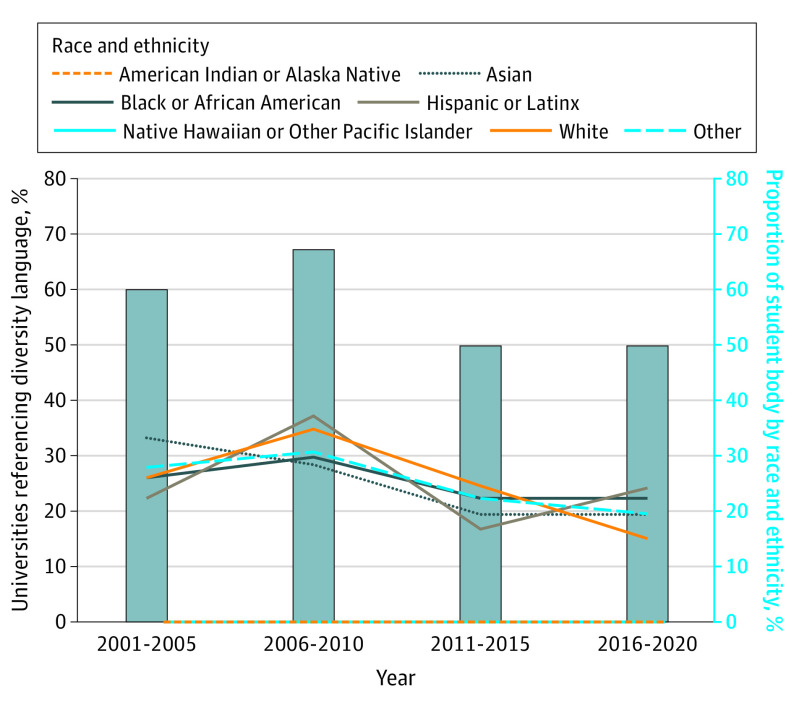
Percentage of Universities That Incorporated Diversity Language in Their Mission Statements and the Racial Distribution of Their Student Bodies Schools established after 2020 were excluded due to incomplete student body demographic data available on websites. Racial categories are in accordance with guidelines provided by the US Office of Management and Budget and the US Census Bureau, and these data are based on self-identification. Other included students who identified as multiple races or ethnicities and students who did not disclose race information.

## Discussion

In this cross-sectional study of 60 US medical schools established from 2000 on, approximately half (45%) of the schools did not reference diversity language in their mission statements. Although newer institutions have the advantage of being less bound by tradition and should be more attuned to the needs of their communities and national health care gaps, our analysis failed to demonstrate significant advancements in these schools over time. Despite increasing recognition of the social accountability of medical schools to serve the health care needs of their communities,^30^ medical schools established within the past 5 years were just as likely (or unlikely) as those established in the early 2000s to include commitments to equity, inclusion, or diversity. This lack of progress becomes more important in light of the US Supreme Court decisions in *Students for Fair Admissions v Harvard*^31^ and *Students for Fair Admissions v University of North Carolina*^32^ that rejected affirmative action, as more schools look to their mission statements to help inform their admissions policies and efforts to diversify the future physician workforce. Our findings suggest that the continued underrepresentation of individuals from historically excluded racial and ethnic groups cannot just be attributed to entrenched institutional policies and power structures.

Several factors have been reported as barriers to increasing diversity in medical schools, including an overreliance on grade point averages and standardized test scores, which have been shown to disadvantage URM applicants^33^; lack of faculty diversity; and insufficient organizational resources and commitment to increasing diversity.^14^ Given the importance of mission statements in communicating identity and purpose to internal and external stakeholders and in driving program priorities and funding,^18,19^ mission statements matter in both what is expressed as well as in what is omitted.

Although some individual medical schools have implemented programs and policies that fulfill their commitment to physician diversity, the presence of mission statements with diversity language of the newly accredited schools was not associated with improved student body diversity. This finding was consistent for both osteopathic and allopathic schools and public and private institutions. Similarly, in an analysis of websites of Canadian medical schools, most institutions included diversity language in response to public calls and government regulations but perpetuated established values and practices, relegating diversity to a peripheral role rather than integrating it as the norm.^34^ Educational psychologists have described a flattening phenomenon whereby diversity is frequently discussed but seldom put into practice.^35^ Mission statements that contain diversity language may be reflecting this phenomenon and be aspirational but not operationalized.

### Limitations

Our study has several limitations. We used publicly available information but did not verify its accuracy with the individual schools. The cross-sectional design does not account for changes in mission statements over time. We used the students’ race and ethnicity classifications as recorded by their institutions; the methodology and accuracy of reporting may differ among schools. We assessed racial and ethnic diversity because these data are most consistently reported but recognize that diversity in the student body requires diversity of multiple factors, including religion, gender identity, socioeconomic status, and physical ability. It is possible that state and federal bans on race-conscious admissions may discourage some institutions in these states from including diversity language in their mission statements and public websites. Our use of broad and varied inclusion terms, such *as cultural competence* and *social responsibility*, was intended to mitigate this potential bias. Finally, public-facing mission statements may not fully capture institutional priorities that are reflected in other documents, such as strategic plans and budgets.

## Conclusions

This cross-sectional study found that in newly established (2000-2020) medical schools, public commitment to diversity and social justice in mission statements was not associated with improved student body diversity. Medical education is a public good, and medical schools should train physicians who can provide care for all communities, particularly historically vulnerable populations where health outcomes lag. There is now consensus that diversity in the national medical student body is essential to fulfilling the social mission of medicine. However, these results suggest that substantial reform is needed in the recruitment and admissions process so that mission statements are not just hollow words.

## Notes

Supplement 1. **eTable.** Codebook of diversity language terms in mission statements

Supplement 2.
Data Sharing Statement
