# Supplementary material for: Diversity in Mission Statements and Among Students at US Medical Schools Accredited Since 2000
Source: JAMA Netw Open. 2023 Dec 14;6(12):e2346916. doi: 10.1001/jamanetworkopen.2023.46916 (PMC10722335; doi:10.1001/jamanetworkopen.2023.46916)
Supplement: Supplement 1. — eTable. Codebook of diversity language terms in mission statements [file jamanetwopen-e2346916-s001.pdf]

Supplemental Online Content

West K, Oyoun Alsoud L, Andolsek K, Sorrell S, Al Hageh C, Ibrahim H. Diversity in mission statements and among students at US medical schools. *JAMA Netw Open*. 2023;6(12).e2346916. doi:10.1001/jamanetworkopen.2023.46916

eTable 1. Codebook of diversity language terms in mission statements

This supplemental material has been provided by the authors to give readers additional information about their work.

© 2023 West K, et al. *JAMA Network Open.*

eTable 1

Codebook of diversity language terms in mission statements

Terms	2001-2005	2006-2010	2011-2015	2016-2020	2021+
Cultural competence	0	0	1	1	1
Different backgrounds	0	0	0	1	0
Disadvantaged	0	0	0	0	0
Disparities	0	0	0	0	1
Diverse/Diversity	1	6	5	6	3
Equality	0	0	0	0	0
Equity	0	0	2	0	0
Ethnicities	0	0	0	1	0
Inclusion	0	1	3	2	1
Indigenous	0	0	0	0	0
Minority	1	0	0	1	1
Multicultural	0	1	1	2	0
Social accountability	0	0	1	0	0
Social Justice	1	0	0	1	0
Social Responsibility	0	0	1	0	0
Socioeconomic	0	1	0	0	0
Underrepresented/ Underserved	0	0	0	2	1
Total	3	9	14	17	8

© 2023 West K, et al. *JAMA Network Open.*
