# Supplementary material for: Diversity in Mission Statements and Among Students at US Medical Schools Accredited Since 2000
Source: JAMA Netw Open. 2023 Dec 14;6(12):e2346916. doi: 10.1001/jamanetworkopen.2023.46916 (PMC10722335; doi:10.1001/jamanetworkopen.2023.46916)
Supplement: Supplement 2. — Data Sharing Statement [file jamanetwopen-e2346916-s002.pdf]

Data Sharing Statement

West. Diversity in Mission Statements and Among Students at US Medical Schools Accredited Since 2000. *JAMA Netw Open*. Published December 14, 2023.
doi:10.1001/jamanetworkopen.2023.46916

Data
Data available: Yes
Data types: Data (not involving human participants)
How to access data: can be obtained from the corresponding author: halah.ibrahim@ku.ac.ae
When available: With publication

Supporting Documents
Document types: None

Additional Information
Who can access the data: Anyone requesting the data.
Types of analyses: For any purpose.
Mechanisms of data availability: After approval.
